# An on Orbit Determination of Point Spread Functions for the *Interface Region Imaging Spectrograph*

**DOI:** 10.1007/s11207-018-1347-9

**Published:** 2018-08-30

**Authors:** Hans Courrier, Charles Kankelborg, Bart De Pontieu, Jean-Pierre Wülser

**Affiliations:** 1 Solar Group, Dept. of Physics, Montana State Univ. Bozeman, P.O. Box 173840, Bozeman, MT 59717-3840 USA; 2 Lockheed Martin Solar & Astrophysics Laboratory, Lockheed Martin Advanced Technology Center, Org. A021S, Bldg. 252, 3251 Hanover St., Palo Alto, CA 94304 USA

**Keywords:** Sun: atmosphere, Instrumentation: high angular resolution, Space vehicles: instruments

## Abstract

Using the 2016 Mercury transit of the Sun, we characterize on orbit spatial point spread functions (PSFs) for the Near- (NUV) and Far- (FUV) Ultra-Violet spectrograph channels of NASA’s *Interface Region Imaging Spectrograph* (IRIS). A semi-blind Richardson–Lucy deconvolution method is used to estimate PSFs for each channel. Corresponding estimates of Modulation Transfer Functions (MTFs) indicate resolution of 2.47 cycles/arcsec in the NUV channel near 2796 Å and 2.55 cycles/arcsec near 2814 Å. In the short (${\approx}\,1336~\mathring{\mathrm{A}}$) and long (${\approx}\,1394~\mathring{\mathrm{A}}$) wavelength FUV channels, our MTFs show pixel-limited resolution (3.0 cycles/arcsec). The PSF estimates perform well under deconvolution, removing or significantly reducing instrument artifacts in the Mercury transit spectra. The usefulness of the PSFs is demonstrated in a case study of an isolated explosive event. PSF estimates and deconvolution routines are provided through a SolarSoft module.

## Introduction

Images from a telescope are often well approximated as a convolution of the real scene with the instrument point spread function (PSF, *e.g.* Hecht, 1987). The PSF describes the response of such an imaging system to a point source of light, altering the perfect mapping of light from the observed object to the image plane of the instrument. The PSF and its Fourier amplitude, the MTF, are widely used for evaluating the performance of a telescope or optical system (*e.g.*, Hecht, 1987; Goodman, 2005; DeForest, Martens, and Wills-Davey, 2009).

The Sun is an extended, high-contrast, and (often) finely structured source in extreme-ultraviolet (EUV) wavelengths (Walker *et al.*, 1988; Golub *et al.*, 1990; Antolin and Rouppe van der Voort, 2012). Observations of such scenes may be subtly smoothed (or blurred) by an instrument PSF, reducing contrast between adjacent bright and dark features. This can affect many different types of measurements, in particular those involving feature photometry (DeForest, Martens, and Wills-Davey, 2009). For example, Shearer *et al.* (2012) found that the PSF scattering wings of the *Extreme-UltraViolet Imager* (EUVI) instrument aboard STEREO-B could significantly alter the diagnostics of temperature and density in coronal holes on the solar disc. Instrument artifacts in solar scenes may be significantly reduced through deconvolution when the PSF is known (*e.g.*, DeForest, Martens, and Wills-Davey, 2009; Poduval *et al.*, 2013) or by blind methods where the scene and the PSF are estimated concurrently (Karovska *et al.*, 1994; Golub *et al.*, 1999). Therefore, a realistic estimate of the instrument PSF is desirable for quantitative interpretation of solar observations.

The *Interface Region Imaging Spectrograph* (IRIS, De Pontieu *et al.*, 2014) is a dual channel solar spectrograph (SG) and slit-jaw imager (SJI) operating in Sun-synchronous orbit since 27 June 2013. As IRIS is a space based observatory, it is not subject to atmospheric distortion, or ‘seeing’ effects. Consequently, all contributions to the IRIS PSFs are due to systematic effects within the observatory *e.g.*, diffraction from the instrument aperture, surface irregularities of the optics (roughness, scratches, dust, figuring error), charge diffusion in the CCDs, and instrument pointing drift and jitter. We infer PSFs along the spatial dimension of the IRIS SG from on orbit observations. Instrumental blurring can then be significantly reduced or removed by deconvolving the inferred PSFs from IRIS SG data.

Solar occultations due to planetary transits or solar eclipses provide a sharply defined shadow that can often be used to characterize the PSF of a Sun-pointed instrument. Weber *et al.* (2007) and Wedemeyer-Böhm (2008) fit PSFs to Mercury transit data observed by the *X-ray telescope* (XRT), *Broadband Filter Imager* (BFI) and the *Solar Optical Telescope* (SOT) onboard the *Hinode* spacecraft. DeForest, Martens, and Wills-Davey (2009) constrained semi-empirical PSFs and measured the effect of stray light in the *Transition Region And Coronal Explorer* (TRACE) extreme-ultraviolet (EUV) telescope using Venus transit observations. More recently, Poduval *et al.* (2013) characterized the diffuse component of the PSFs for the *Atmospheric Imaging Assembly* (AIA) EUV telescopes on board the *Solar Dynamics Observatory* (SDO) spacecraft using a lunar occultation of the Sun.

We use the May 2016 Mercury transit to characterize on orbit PSFs for the IRIS SG. While Mercury’s limb (when occulting the Sun) provides a sharply defined edge from which a spatial component of the SG PSF may be derived, the region in Mercury’s shadow is devoid of any significant spectral information. Therefore, we confine our attention to blurring in the spatial direction only (parallel to the SG slit) and assume that the PSF is effectively invariant across the passband in each SG channel.

The structure of the paper is as follows: In Section 2 we describe IRIS, the data, and processing required for our analysis. Section 3 describes model PSFs for the far ultraviolet (FUV) and near ultraviolet (NUV) SG channels, and the semi-blind deconvolution process we use to derive NUV PSF estimates. In Section 4 we present these estimates and discuss their applications with representative deconvolved data. We conclude by summarizing the characteristics of our PSF estimates and present deconvolved spectra in Section 5.

The PSFs determined here are distributed via SolarSoft (SSW, Freeland and Handy, 1998) with a deconvolution routine, iris_sg_deconvolve.pro, suitable for direct application to IRIS SG Level 2 data.

## Instrument and Data Selection

The IRIS instrument uses a Czerny–Turner style spectrograph and a slit-jaw imager (SJI) to obtain solar images and spectral information over a range of ultraviolet (UV) wavelengths. The SG and SJI instruments are fed by a single 19 cm Cassegrain telescope (Podgorski *et al.*, 2012). A slit prism at the focus of the telescope separates incoming light into three light paths: SG FUV (short, 1332 – 1358 Å, and long, 1389 – 1407 Å, channels), SG NUV (2783 – 2835 Å channel), and SJI channel. Spectra and images are obtained by four $2061 \times 1056$ pixel CCDs. The field of view (FOV) of the SG slit is $0.33'' \times 175''$. SG rasters are created by scanning the active secondary mirror of the Cassegrain telescope, for a maximum raster FOV of $130'' \times 175''$. Each 13 μm pixel of the SG CCDs subtends $0.167''$ spatially and 12.8 mÅ (FUV) or 25.5 mÅ (NUV) spectrally.

Level 2 IRIS data are available for download from the Lockheed Martin Solar and Astrophysics Laboratory (LMSAL: https://iris.lmsal.com/data.html) and are considered the standard science product. The Level 2 data are 32-bit floating point numbers. Processing IRIS data to Level 2 includes removing overscan rows, reorienting images to common axes, removing dark currents and pedestal, flat fielding, applying geometric and wavelength calibrations, mapping images to a common plate scale, subtracting FUV background, and recasting images into rasters (SG) and time series (SJI) (De Pontieu *et al.*, 2014). The level 2 SG data may further be subdivided into spectral regions, or “windows,” corresponding to a spectral line list that reads out only a portion of the CCDs at the time of observation. The spectral regions are denoted by the emission or photospheric reference line wavelength they encompass (*e.g.* 1336, 1394, 1403, 2796 and 2814 Å; De Pontieu *et al.*, 2014). IRIS observed the Mercury transit using a full readout for 15 s exposures and a small linelist otherwise (see *e.g.* Table 1).

**Table 1:** IRIS Mercury transit observations.

Data set	Obs. period UTC	Exposure		Max. occultation		Notes
		# of exps.	len. (s)	Index	UTC	
1	10:50:08 – 11:34:59	514	4	287	11:15:14	Sit & stare, SL
2	11:44:39 – 12:23:44	448	4	226	12:04:24	Sit & stare, Rot. track, SL
3	12:33:44 – 13:06:00	600	2	380	12:54:12	Sit & stare, Rot. track, SL
4	13:16:31 – 13:54:53	140	15	74	13:36:57	Sit & stare, Rot. track, FR
5	14:04:46 – 14:48:34	502	4	293	14:30:23	Sit & stare, Rot. track, SL
6	14:59:55 – 15:26:08	96	15	62	15:17:02	Raster, FR
7	15:36:23 – 16:20:57	512	4	264	15:59:24	Sit & stare, Rot. track, SL
8	16:31:03 – 17:10:31	144	15	72	16:50:55	Sit & stare, Rot. track, FR
9a	17:25:48 – 18:00:41	400	4	0	17:25:48	Raster, SL, †
9b				1	17:25:53	
9c				2	17:25:58	
9d				4	17:26:09	
9e				6	17:26:19	
9f				8	17:26:30	
10	18:10:41 – 19:00:57	576	4	337	18:40:09	Sit & stare, W1

SL: Small line list: 1336 (C ii) and 1403 (Si iv), NUV: 2796 (Mg ii k), and 2814 Å.

FR: Full readout. To maintain consistency between data sets, we analyze only the portions of the NUV spectra that overlap with the windows listed in SL from the full readouts.

† Raster motion closely matched that of Mercury at the start of data set 9, therefore maximum occultation was imaged multiple times in this data set.

**Figure 1:**
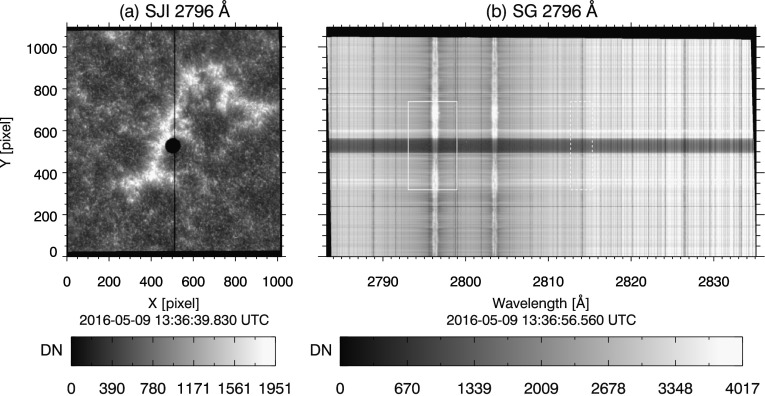
Panel (**a**), IRIS 2796 Å slit-jaw image of Mercury transit. Mercury is the dark shadow near center, the *dark vertical line* through the center of the image is the spectrograph slit. Panel (**b**), corresponding full NUV spectrograph readout. Mercury’s shadow is the dark horizontal band across spatial pixels ${\approx}\,490$ to 570. *Thin horizontal dark lines* near spatial pixels 240 and 775 are fiducial marks on the slit. *Solid white box* outlines data analyzed for the 2796 Å window; *dashed box* is the same for 2814 Å window. The vertical extent of both boxes is sized to exclude the fiducials.

On 09 May 2016, as seen from Earth, Mercury transited across the solar disc from ${\approx}\,10{:}50\,\mbox{--}\,19{:}00~\mbox{UTC}$, moving from east to west. IRIS imaged the entire transit in ${\approx}\,50$ minute increments, the length of time required for Mercury to travel across the SJI FOV. Approximately 10 minutes of observing time was missed between each increment, while the spacecraft changed pointing to keep Mercury in the SJI field of view. The SG slit was oriented parallel to the solar north–south axis for the duration of the transit, so that Mercury crossed the slit at least once during each pointing. Figure 1 panel (a) shows a typical 2796 Å SJI image during the transit. The SG slit is visible as a dark vertical line, passing through the shadow of Mercury near center of Figure 1(a).

From the 10 sets of observations IRIS made during the transit, only a handful of spectra were suitable for estimating the SG PSF. Spectra were selected in which the signal-to-background ratio (SBR) was large enough so that both limbs on either side of Mercury were sharp and well defined. The observing period, exposure length, location of maximum occultation, and observing notes are listed in Table 1 for each of the 10 SG data sets. To minimize the contribution of Mercury’s motion to the PSF, we selected only the spectra from each data set where the occultation by Mercury’s shadow was maximized over the slit. Except for data set 9, Mercury crossed the SG slit once in each data set. In data set 9, the raster motion tracked Mercury for several frames at the beginning of the observation, resulting in six occurrences of maximum occultation.

The selection criteria were met by data sets 2 through 9 in the NUV channel. Data sets 1 and 10 were ignored because the shadow of Mercury was partially off the solar disc when imaged by the SG. For the off-disc portion of Mercury, SBR is insufficient to present a well-defined limb in the NUV spectra. Figure 1 panel (b) shows the full readout NUV CCD spectrum from data set 4; residual intensity from the instrument PSF is clearly seen where Mercury’s shadow crosses the Mg ii doublet. From the full NUV readouts (data sets 4, 6, and 8), we select only the spectral regions that correspond to the 2814 Å and 2796 Å windows (see Section 2.1) to maintain consistency across all data sets. Of the NUV windows, 14 suitable spectra were obtained for both the 2814 Å and the 2796 Å windows.

For the FUV windows (1403, 1394, and 1336 Å), only the three 15 s exposures (data sets 4, 6, and 8) from the 1394 Å and 1336 Å windows had sufficient SBR to present well-defined limbs of the shadow of Mercury. Fewer data sets in FUV spectra meet the selection criteria because quiet Sun and active region intensity in the FUV (*i.e.*, C ii, Si iv, and O iv, Vernazza and Reeves, 1978; Cook *et al.*, 1995) is weaker than that of the NUV (Mg ii and NUV continuum, Morrill and Korendyke, 2008; Staath and Lemaire, 1995). For this reason, and because SG throughput decreases with wavelength (De Pontieu *et al.*, 2014), SBR is sufficient only in the data sets with the longest (15 s) integration times. We also note that in two of the three FUV data sets, the north limb of Mercury is over a region of bright plage (see, *e.g.*, Figure 3). This results in significantly greater contrast at the north limb compared to the south limb.

We modify the NUV and FUV spectra selected above for input to our semi-blind deconvolution routine. Preparation of the data differs slightly between the NUV and FUV cases, largely due to the lower SBR of the FUV data. In the following subsections we first describe reduction of the NUV spectra, then we describe the differences in this process for the FUV spectra.

### NUV Data Reduction

IRIS SG images contain fiducial markings to aid in the geometric alignment of the spectra. These markings are gaps in the slit, and appear as two dark horizontal bands in the spectra (see, *e.g.*, Figure 1 panel (b) near spatial Y pixels 240 and 775). Since the slit prism is placed after the telescope optics, the images of the fiducial markings formed on the SG CCDs are not affected by aberrations inherent to the telescope. The PSF in the region of the fiducial markings is therefore narrower. To prevent biasing the PSF estimate, we cropped the fiducial markings from each transit spectrum. A buffer region of several pixels adjacent to each fiducial is removed, the size of which varies so that the shadow of Mercury is centered in the image frame.

We reduce the data to 1D by spectrally summing the transit images. There are two advantages to performing this sum; 1) our task is reduced to finding a PSF in one dimension instead of two, and 2) the signal-to-noise ratio (SNR) is improved for the summed data set. In the NUV the presence of continuum in the spectrum contributes to the overall signal level, so we include the entire window in the sum. Data analyzed in the NUV windows is outlined by dashed (2814 Å) and solid (2796 Å) boxes in Figure 1 spanning 103 and 232 spectral pixels, respectively. There are two underlying assumptions in our choice to sum over wavelength. The range of reflection angles across the spectral window is small, so we assume that any variation in the PSF with respect to wavelength over the range of a spectral window is small enough to be ignored. This assumption is supported by the nearly identical PSFs in each of the two FUV and NUV windows, estimated independently by our Fourier model in Section 3 and our results in Section 4. The second assumption is that dispersion is oriented precisely along the pixel rows (*i.e.* the x-axis, or dispersion axis, of the spectra). This applies only to IRIS Level 1.5 data and higher, in which the small instrumental misalignment of the spectrum to the SG CCDs has been corrected for (De Pontieu *et al.*, 2014). We have also verified by hand that the fiducial markings are aligned precisely row-wise within each spectral window.

As part of the Level 1.5 processing, the IRIS spectra are warped by a second order polynomial so that the spatial and spectral dimensions are rectilinear with respect to the CCD pixel grid (Jaeggli, 2016). The geometric correction varies in time and space. A side effect of the image warping (*i.e.* geometric correction) is that sub-pixel shifts result in varying attenuation of high spatial frequencies. Since the correction is small, the offset from the original to the corrected grid of pixels varies slowly across the CCD. For the worst case of a half-pixel shift, the Nyquist frequency is completely lost. In the spatial dimension, the result is a periodic blurring. The phase of this periodic blurring varies image-to-image due to temporal alignment variations in the instrument. By summing along the spectral axis, we have averaged the best and worse cases of this blurring in each image.

#### Estimation of Mercury’s Limb

**Figure 2:**
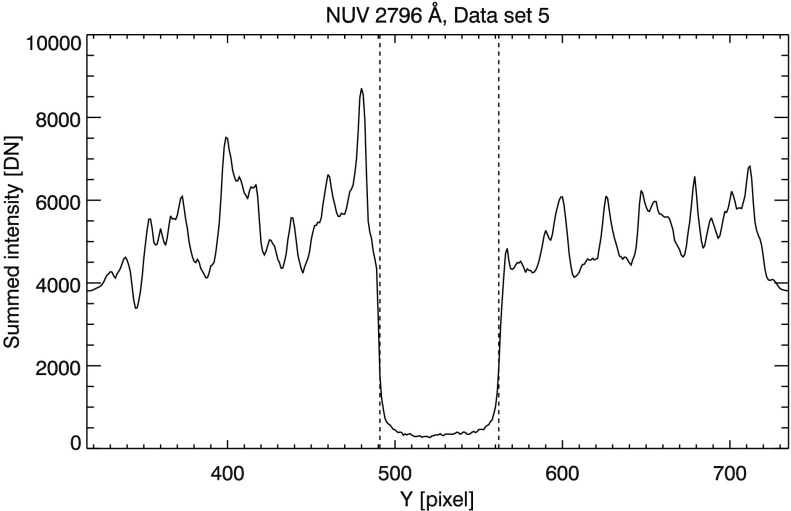
Reduced data from set 5. *Vertical dashed lines* mark Mercury limbs. Data are summed over the 232 spectral pixels in this 2796 Å window, the horizontal extent of the solid white box in Figure 1 panel (b). Fiducial markings are removed by cropping data.

The reduced version of the solid white box in Figure 1 panel (b) is plotted in Figure 2; the vertical dashed lines mark the locations of Mercury’s limbs. The limbs are located by finding the spatial locations of maximum and minimum derivatives of the reduced spectra intensity in a region that encompasses the entire shadow of Mercury. The derivative is computed using centered differencing,

1 $$ \frac{\mathrm{d}I}{\mathrm{d}y} \bigg|_{y=y_{i}} = \frac{I(y_{i+1}) - I(y_{i-1})}{y_{i+1} - y_{i-1}}, $$

where $I(y)$ is the summed intensity at pixel $y$ in the reduced data. Errors in limb location of at least ${\pm}\,1$ pixel are expected from discretization; noise in the data may increase this uncertainty. Only approximate locations of the limbs are needed to constrain the semi-blind deconvolution described in Section 3.2. Except data sets 5 and 7, we find the locations of the limbs to be consistent between the two NUV windows using this method. For these two data sets, the north limb is displaced one pixel northward in the 2796 Å window when compared to the 2814 Å window. From the limb locations we find that Mercury’s shadow spans 72 – 73 pixels, or an angular diameter of $12\,\mbox{--}\,12.2''$. For comparison, the angular diameter of Mercury when viewed from Earth in May is typically $12''$ and was estimated to subtend $12.1''$ during the time of the transit (U.S. G.P.O., 2015).

### FUV Data Reduction

The FUV data reduction proceeds in a similar fashion as the NUV, but with notable modifications described below.

**Figure 3:**
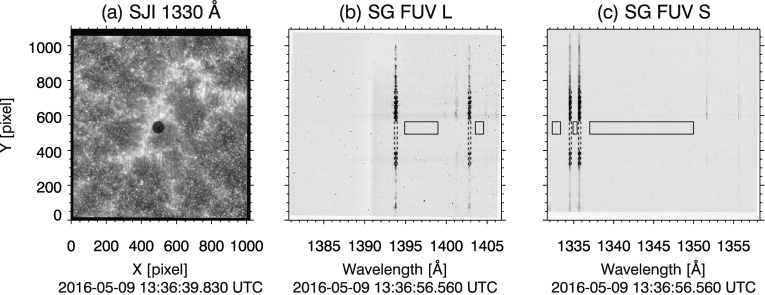
(**a**) 1330 Å SJI context image of Mercury transit. (**b**) and (**c**), full readout of data set 4 in the two FUV channels 1394 Å and 1336 Å windows, respectively. Only the spectral lines enclosed by the *black dashed boxes* are analyzed to maximize SBR. Dark subtraction error is estimated by no-signal pixels in Mercury’s shadow, enclosed by the *black solid boxes*. Intensity is logarithmically scaled in all panels; SG images are displayed in inverse gray scale.

The continuum signal is absent in the FUV. The pixels between the bright emission lines contain only background and readout noise. We therefore sum over only the bright FUV spectral pixels outlined by the dashed boxes in Figure 3 panels (b) and (c).

Contrast is insufficient to locate the limb using the derivative method. We select the limb locations of the 2814 Å window as the reference for the FUV data sets. Using the co-alignment performed between spectral windows during Level 2 processing, the limb locations are transferred to the FUV windows.

We find the mean intensity in Mercury’s shadow is negative in several of the data sets. This could be a result of small errors in the dark subtraction, which are amplified by spectral summing. To correct this, we estimate the offset error by computing the mean per-pixel intensity in no-signal regions of Mercury’s shadow in the FUV data sets. These regions are outlined by solid black boxes in (b) and (c) of Figure 3. We find offset error values that range from −0.03 to 0.06 DN (1394 Å) and −0.04 to $-0.18~\mbox{DN}$ (1336 Å). We correct these intensity errors by adding a DC offset to the FUV data sets. The value of the DC offset is such that the mean per-pixel intensity in the regions outlined in Figure 3(b) and (c) is zeroed.

## PSF Estimation

We estimate the IRIS SFG PSFs using a modified blind iterative deconvolution (BID) technique, in contrast to the parameterized and semi-empirical methods referenced in Section 1. BID typically refers to a technique first developed by Ayers and Dainty (1988); however, it can refer to any image restoration technique in which both the un-degraded image and its degrading function (typically the PSF) are unknown. BID techniques are most often used when *a priori* knowledge is limited to only non-negativity of the images. For example, the Ayers and Dainty (1988) method was subsequently used to derive PSFs and deconvolve images from *Skylab* and *Yohkoh* data (Karovska *et al.*, 1994), and to estimate the on orbit PSF for the TRACE instrument (Golub *et al.*, 1999).

Determining PSFs using BID techniques presents an ill-posed problem. Parameterizing the PSF may be considered a more robust approach (DeForest, Martens, and Wills-Davey, 2009); however, the BID method can be improved by incorporating *a priori* information (Fish *et al.*, 1995) resulting in the ‘semi-blind’ description of the modified BID technique. We find that the IRIS Mercury transit data lends itself well to this particular type of analysis. In much of our data, solar features immediately behind Mercury’s limb are not uniformly illuminated (see, *e.g.*, either side of Mercury’s shadow in Figure 2). As a result, the data are not well approximated by a step function precluding a similar analysis such as performed by Weber *et al.* (2007) and Wedemeyer-Böhm (2008). DeForest, Martens, and Wills-Davey (2009) and Poduval *et al.* (2013) used scattered light in the interior shadows of occultations by Venus and the Moon to semi-empirically constrain stray light PSFs for the TRACE and the AIA assembly aboard SDO. This form of analysis is largely concerned with scattering wings so that detailed information on the core structure of the PSFs is excluded. Since we have no *a priori* knowledge of the IRIS PSF, we find BID is better suited to our purposes since we can estimate the entire PSF from the same data set. Knowledge of the core structure of the PSF allows us to investigate the resolution of the SG (Section 4) and ultimately may influence the fine structure that we observe in the IRIS data.

**Figure 4:**
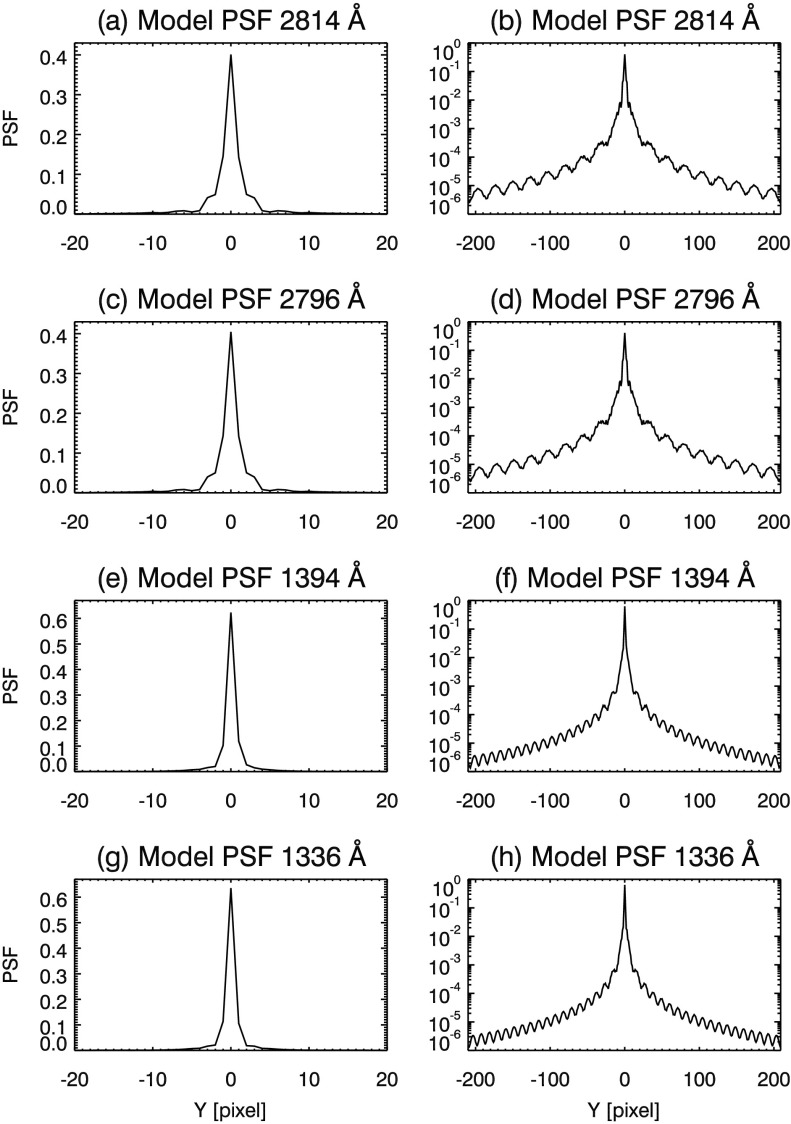
Diffraction limit model PSFs for the IRIS Cassegrain telescope. Top panels show the core (**a**) and wings (**b**, log scale plot) of the NUV 2814 Å PSF. Remaining panels depict the same for the (**c**, **d**) NUV 2796 Å, (**e**, **f**) FUV 1394 Å, and (**g**, **h**) 1336 Å windows.

A further advantage of a BID approach is that our PSF model is not constrained by a small number of parameters. IRIS operates close to the diffraction limit so that the core of the SG PSF may show significant structure, such as side lobes or asymmetry (see, *e.g.*, Figures 4 and 7). The parameterized models referenced earlier cannot reproduce either of these structures, so by using BID we are free to find features that we may not have guessed in advance. This allows us to derive a PSF that is consistent with the data at hand.

For the above reasons, we feel that a BID method of estimating the IRIS PSFs is advantageous over a parameterized model. We base our BID on the Richardson–Lucy algorithm (see Equation 3), as this method has proven robust in the presence of noise (Fish *et al.*, 1995). In the remainder of this section we describe initialization (and how we include *a priori* information), the iterative routine, and stopping criterion for our semi-blind deconvolution method.

### Initial Guesses

Before we begin the process of deconvolution to determine the PSF, we must prepare initial guesses both for the ideal deconvolved data and for the PSF. In both cases, we will make use of prior information. For that reason, we describe the deconvolution as *semi*-blind.

#### Mercury’s Shadow

**Figure 5:**
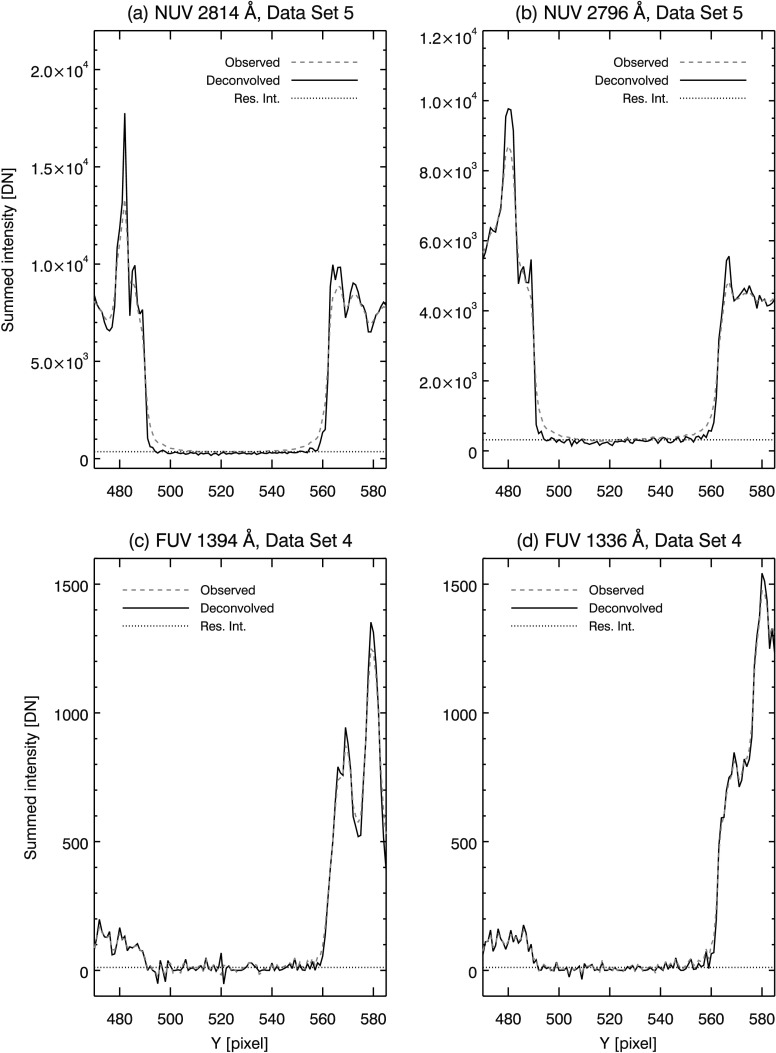
Model PSFs deconvolved from reduced data (*solid curves*), with original overplotted (*dashed curves*). Panel (**a**) shows NUV 2814 Å window, (**b**) NUV 2796 Å, (**c**) FUV 1394 Å, and (**d**) FUV 1336 Å. Mean residual intensity in Mercury’s shadow after deconvolution is plotted as the *dotted curve* in each panel.

Since Mercury is opaque, Mercury’s shadow during the solar transit will contain no UV radiation. Ideally, the limb of Mercury should have a ‘hard’ edge in the SG spectra, where the signal drops abruptly to zero in the shadow region. In the observed spectra, the SG PSF softens this edge and causes residual intensity to appear in the shadow of Mercury (see, *e.g.*, Figures 1, 2, and 5), indicating some level of scattered light is present in all spectral windows.

As an initial guess for the NUV deconvolved scene, we modify the reduced data set by setting all pixels to zero between limb locations. The two pixels at the limbs are not set to zero, to allow for subpixel location of the limb. Since the semi-blind deconvolution is a multiplicative process of the initial guess (see, *e.g.*, Equations 2 and 3), any pixel that is initially set to zero will remain unchanged after each iteration. This initial guess therefore enforces the prior knowledge that the scene should contain no light in the shadow of Mercury.

In the FUV, low SNR results in some negative intensity values. Convergence of the Richardson–Lucy algorithm is neither desirable nor possible in this case (Lucy, 1974). To counter this, we add a positive DC offset to the FUV reduced data. The pixels in Mercury’s shadow are then forced to the value of the DC offset after each iteration, rather than zero as in the NUV case discussed above. The magnitude of the offset is arbitrary; it only needs to be large enough so that negative noise pixels are not ‘clipped’ at zero. We choose a value of $10^{3}~\mbox{DN}$, which is ample margin to prevent clipping. The offset is subtracted after deconvolution to yield an unbiased estimate of the deconvolved scene.

#### Model PSFs

Using the theory of Fraunhofer diffraction (*e.g.* Goodman, 2005, p. 74, or similar references), we calculate the diffraction pattern of the Cassegrain telescope aperture at the emission line wavelength for each of the four spectral windows. We reduce this 2D pattern to a 1D spatial PSF by summing along the axis parallel to the dispersion direction of the instrument. Spatial PSFs for the NUV and FUV windows are plotted in Figure 4. The log plots of Figure 4 clearly show side lobes in the wings of the model PSFs. The side lobes are a result of the fringes of the diffraction pattern from the telescope entrance aperture, including central obscuration and spiders. For the remainder of this report, we define the core of the PSF as the radius extending to five pixels on either side of the individual PSF peak value. The output of the semi-blind deconvolution depends on the initial PSF choice, and in Section 4 we consider an alternate initialization.

Deconvolving the model PSFs from the reduced data gives an indication of the SG performance compared to the diffraction-limited case. In Figure 5, model PSFs are deconvolved from the reduced data and overplotted (solid curves) on the reduced data (dashed curves). The iterative Richardson–Lucy algorithm is used to perform the deconvolution. This method of deconvolution corrects large deviations from the true scene in relatively few iterations; further iterations result in small corrections that slowly tend to match the statistical fluctuations in the observed scene (*i.e.*, noise, *e.g.* Lucy, 1974). For this reason, a cost function is typically used to truncate iterations. Often this takes the form of a $\chi ^{2}$ goodness of fit between the observed scene and a forward model consisting of the deconvolved scene re-convolved with the PSF. For our purposes, we use a modified form of the Kullback–Leibler (KL) divergence (Bertero *et al.*, 2009) as the cost function, so that negative pixels may be handled in the same way as Section 3.1.1. To prevent fitting to noise, we truncate iterations when the derivative of the KL divergence is ≈ zero. Experimentally, we find 50 iterations in the NUV and 9 – 10 iterations in the FUV windows are sufficient to meet this criterion.

In the NUV channel, the effect of deconvolution is evident in panels (a) and (b) of Figure 5. Residual intensity is clearly greater than zero in these panels, indicating not enough energy is present in the wings of the NUV model PSFs. The core of the NUV model PSFs also needs correction, as prominent ’shoulders’ are still evident at Mercury’s limb after deconvolution. In panels (c) and (d) of Figure 5, deconvolution of the diffraction-limited PSF results in little change for the two FUV spectra. Some sharpening of the north limb is evident (*e.g.* near pixel 560 in panels (c) and (d) in Figure 5); however, residual intensity (dotted line, refer to Section 3.2) remains slightly greater than zero in the shadow of Mercury. Mercury’s limb is sharpened, and scattered light is reduced in both SG channels by deconvolving the model PSFs; however, Figure 5 clearly shows that the model PSFs are inadequate at describing the SG performance in the NUV channel. The effectiveness of the FUV model PSFs are more difficult to discern due to the lower SBR in this channel.

### Semi-blind Deconvolution

PSFs were estimated using an iterative blind deconvolution routine to simultaneously estimate the PSF and the deconvolved image. We consider our implementation to be a semi-blind method, since *a priori* knowledge of both the diffraction-limited form of the instrument PSF and the complete opacity of Mercury are incorporated into the first iteration of the routine by the initial guesses described previously. The deconvolution routine consists of alternating applications of Richardson–Lucy deconvolutions (Richardson, 1972; Lucy, 1974), expressed as

2 $$\begin{aligned} g^{i+1}(y) =& \biggl[ \biggl(\frac{c'(y)}{g^{i}(y) * f^{i}(y)} \biggr) * f^{i}(-y) \biggr] \cdot g^{i}(y), \end{aligned}$$

3 $$\begin{aligned} f^{i+1}(y) =& \biggl[ \biggl(\frac{c'(y)}{f^{i}(y) * g^{i+1}(y)} \biggr) * g^{i+1}(-y) \biggr] \cdot f^{i}(y) , \end{aligned}$$

where $g(y)$ is the PSF, $f(y)$ is the deconvolved image, $c'(y)$ is the original (detrended) degraded image, $y$ is spatial position in IRIS pixels, $i$ is iteration number, and ∗ is the convolution operator (Fish *et al.*, 1995). One iteration consists of one evaluation of Equation 2 to find the next PSF estimate, $g^{i+1}(y)$, followed by one evaluation of Equation 3 to find the next deconvolved image, $f^{i+1}(y)$ (Holmes, 1992). Integration of Equation 3 shows that the total flux is conserved. Conservation of flux is enforced in Equation 2 by re-normalizing $g^{i}(y)$ after each iteration. Iterations are started with the guesses from Section 3.1.1 and Section 3.1.2 for $f^{0}(y)$ and $g^{0}(y)$, respectively.

The convolution operations in Equations 2 and 3 are performed in Fourier space for computational efficiency. To reduce contamination from edge effects, we detrend the reduced data. For the left edge of $c(y)$, we write this as

4 $$ c'(y)=\left \{ \textstyle\begin{array}{l@{\quad}l} \mu \cos ^{2} [\frac{\pi y}{2a} ] + c(y)\sin ^{2} [\frac{\pi y}{2a} ], & 0 \leq y \leq a, \\ c(y), & y>a, \end{array}\displaystyle \right . $$

where $\mu $ is the mean of the two endpoints of $c(y)$ and $a$ is the number of pixels the smoothing extends from the edge of the data. A similar treatment is applied to the right edge (see, *e.g.*, Figure 2). We set $a=30$, the geometric mean of the PSF half width (five pixels) and the length of data from the left edge to Mercury’s limb (${\approx}\,175$ pixels). To prevent a symmetric bias in the data, we pad $c'(y)$ to twice its original length with the value of $\mu $ and zero pad $g^{i}(y)$ to match.

### Stopping Criterion

**Figure 6:**
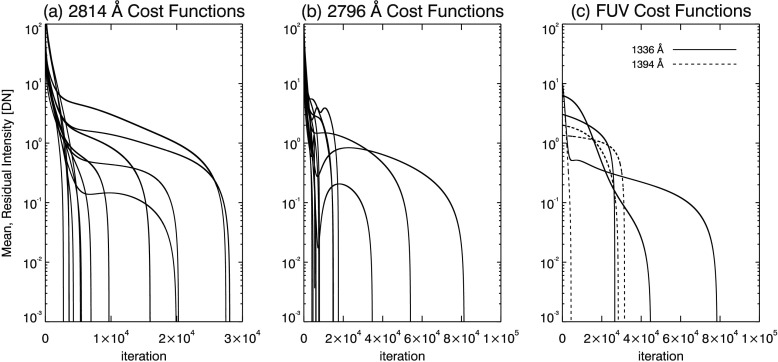
Log plot of cost functions for the (**a**) NUV 2814 Å, (**b**) NUV 2796 Å, and (**c**) FUV 1336 (*solid*) and 1394 Å (*dashed*) windows. The cost function is a measure of residual intensity in Mercury’s shadow. Iterations are truncated when the cost function reaches zero.

Since the Richardson–Lucy process is an iterative approximation to deconvolution (Lucy, 1974), iterations are truncated when a stopping criterion is met. The stopping criterion is typically derived from a cost function that quantifies the difference between the observed data and a forward model formed from the deconvolved function and the PSF. This function may take different forms depending on the application (*e.g.*, Richichi, 1989; Fish *et al.*, 1995; Bertero *et al.*, 2009, and the references therein). For our cost function we choose the mean value of residual intensity in the shadow of Mercury, since *a priori* it is known that this value is zero in the ideal case. We write this function as

5 $$ \mathcal{C}^{i} = \frac{1}{ [N-n+1 ]}\sum _{k=n}^{N}\mathcal{F}^{-1} \biggl[ \frac{\mathcal{F}[c(y_{k})]}{\mathcal{F}[g^{i}(y_{k})]} \biggr] , $$

where ℱ ($\mathcal{F}^{-1}$) is the (inverse) Fourier transform, $c(y_{k})$ is the original degraded data, and $k$ runs only over those pixels between the south ($n$) and north ($N$) limbs of Mercury’s shadow. The deconvolution is performed as division in Fourier space, so that non-negativity is not inherently enforced. This results in an unbiased estimate of the mean shadow intensity. Iteration of the semi-blind deconvolution is truncated when $\mathcal{C}^{i} < 0$ so that the previous PSF, $g^{i-1}(y_{k})$, is the best estimate that does not over-deconvolve the data. In Figure 6 we plot cost functions for all data sets. In every case the cost function reaches a zero-crossing, so that the moment of convergence is well defined.

## Results and Discussion

**Figure 7:**
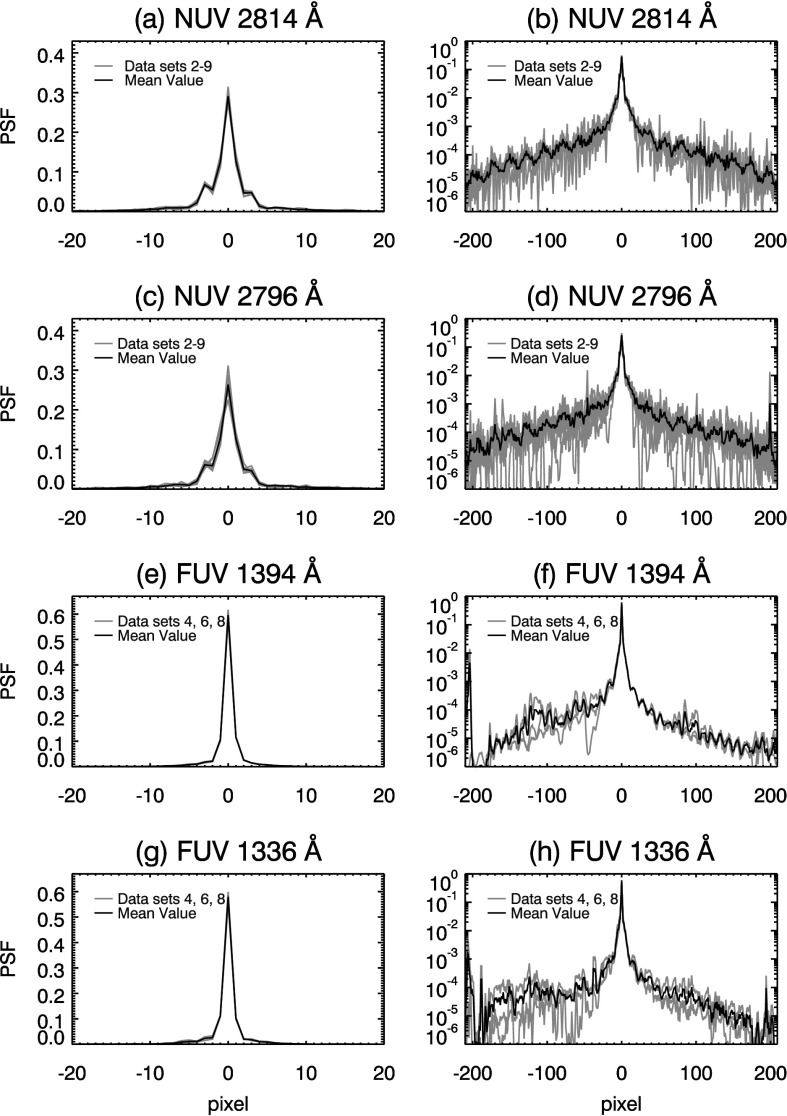
PSF estimates from the semi-blind Richardson–Lucy routine for the (**a**, **b**) 2814 Å, (**c**, **d**) 2796 Å, (**e**, **f**) 1394 Å, and (**g**, **h**) 1336 Å observation windows. *Gray curves* represent the estimate(s) from data sets 2 – 9 for the NUV and 4, 6, and 8 for the FUV windows. The mean value PSF is overplotted as a *black curve* in each panel. Variation in the left wing of (**f**) and (**h**) results from low contrast in quiet Sun area south of Mercury, see text for details.

Figure 7 displays normalized PSFs for the selected data sets (light gray curves in plots) of the NUV and FUV spectral windows. In each panel of Figure 7, the black curve is the mean of PSFs in that spectral window. We take the mean as the best PSF estimate for the two NUV windows, since it preserves normalization while reducing the noise in the wings of the PSFs. We note that the two NUV PSF estimates show similar core (panels (a) and (c), Figure 7) and wing structures (panels (b) and (d), Figure 7) despite being derived from different spectral windows. The similarities between the NUV PSF estimates conforms to our expectation that the PSF should not change significantly over a short wavelength range. We assume that any differences between the two NUV PSFs are due to random error.

For the FUV 1394 Å and 1336 Å windows (panels (e) and (g) of Figure 7, respectively), the mean PSFs also display similar core structure. In data sets 4 and 6, the southern limb of Mercury is positioned over a region of quiet Sun. Lower solar emission in this region reduces the contrast of this limb in these two data sets. In Figures 7 (f) and (h), this manifests as significant variation in the left wing of the FUV PSFs. To remove the uncertainty, we assume the FUV PSF wings are symmetric. We replace the left wing of the mean PSF in both FUV spectral windows ($\mbox{pixels} < -5$, panels (f) and (h) Figure 7) with a mirrored version of its right wing ($\mbox{pixels} > 5$), then renormalize the result to unity (see, *e.g.*, (f) and (h) in Figure 14).

Comparing (a – d) of Figure 7 to the same panels in Figure 4, we find that our NUV PSF estimates have more distinct, asymmetric side lobes than the diffraction-limited case. The wings of the NUV PSF estimates are also flatter, which indicates more light is scattered in the NUV channel than the diffraction-limited case. Comparing panels (e – h) of Figure 7 and Figure 4 we do not find side lobes in the FUV PSF cores; however, wing structure is very similar in both cases.

Table 2 lists the wing and core energies for the diffraction limit and PSF estimates. The error ranges reported in Table 2 reflect uncertainties in dark subtraction. If the dark signal is overestimated, then after subtraction the signal will be low, and the method will underestimate the core width of the PSF. Low signal also reduces the residual intensity in Mercury’s shadow, causing the cost function to truncate iterations early. In general, as the iterations proceed, flux migrates from the PSF core to the tails. Consequently, an overestimate in dark signal results in an underestimate in scattered light. The inverse is true if the dark signal is underestimated. In the NUV channel, dark subtraction uncertainty is ${\pm}\,0.1~\mbox{DN}$ per pixel (Wülser *et al.*, 2018). For the FUV channels, lower SBR means that the offset error must be asymmetrically bounded so that residual intensity in Mercury’s shadow is not reduced below zero (clearly an unphysical situation). For an underestimate of dark subtraction, we use the greater of the maximum magnitude of offset error noted in Section 2.2 or the per-pixel residual intensity in Mercury’s shadow in each FUV channel. There is no bound for overestimating dark subtraction, so we may use the maximum magnitude of offset error from Section 2.2.

**Table 2:** PSF energy distribution.

Window (Å)	Diff. model		PSF estimate		
	Core	Wings	Core	Wings	PLSR
2814	0.898	0.102	0.815 ± 0.003	0.185	0.725 ± 0.003
2796	0.899	0.101	0.789 ± 0.010	0.211	$0.652^{+0.008}_{-0.010}$
1394	0.954	0.046	$0.951^{+0.003}_{-0.078}$	0.049	$0.975^{+0.025}_{-0.098}$
1336	0.957	0.043	$0.939^{+0.015}_{-0.041}$	0.061	$0.928^{+0.066}_{-0.052}$

We estimate total dark subtraction uncertainty as a simple DC offset to the SG spectra. PSFs are recomputed for the extrema of dark subtraction offset to arrive at the uncertainties listed in Table 2. The asymmetry in FUV offset error carries through to the listed FUV uncertainty. The last column of Table 2 is computed by direct analogy to Strehl ratio. We call this *pixel-limited Strehl ratio* (PLSR), since integration of the peak intensity over the central pixel reduces the peak value of each PSF. Uncertainty in PLSR is estimated in a similar fashion to the core energy. We note that, for an underestimate of dark subtraction error, PLSR is reduced and *vice versa* for an overestimate.

**Figure 8:**
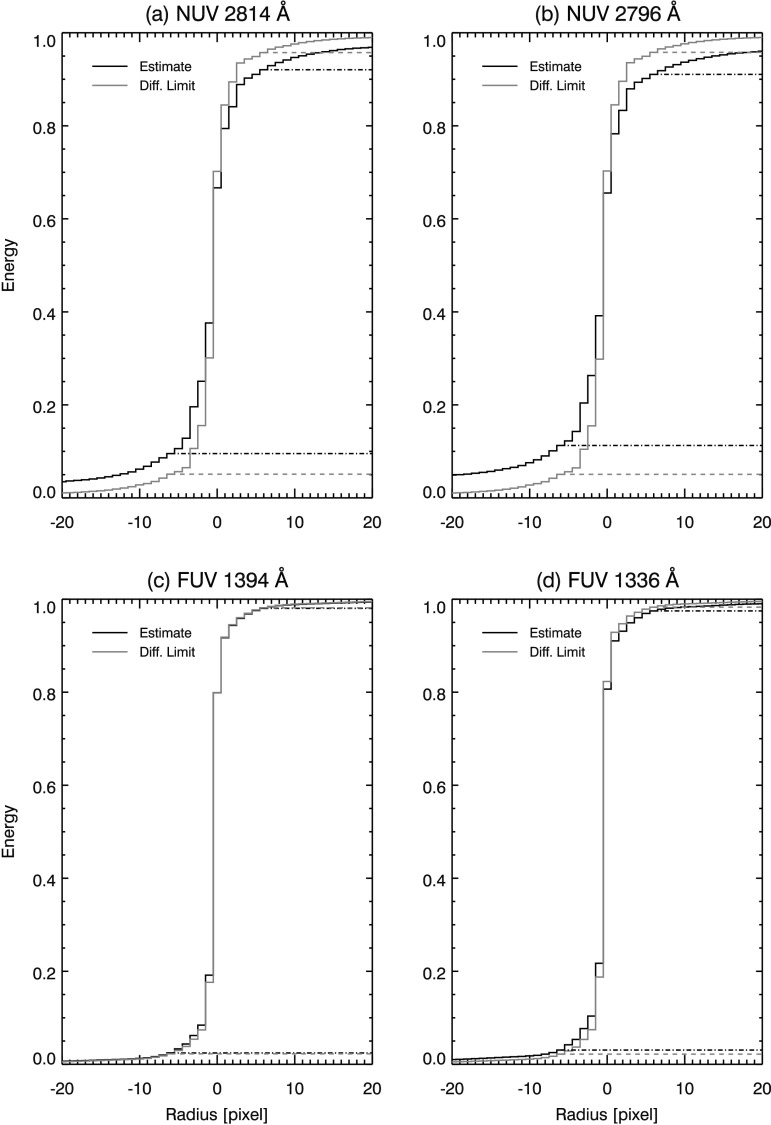
Cumulative total energy for the NUV (**a**) 2814 Å, (**b**) 2796 Å, FUV (**c**) 1394 Å, and (**d**) 1336 Å windows. Total energy is calculated between *horizontal line pairs*, *solid* for diffraction limit PSFs and *dashed* for PSF estimates. NUV plots (**a** and **b**) show similar asymmetric energy distributions.

As another measure of how energy is distributed between the scattering wings and core, we calculate the cumulative total energy of the NUV PSF estimates. Dash (diffraction-limited) and dash-dot (PSF estimate) horizontal lines demarcate the energy contained in the core of the PSF for each panel in Figure 8. Figure 8 also shows that the asymmetry of the NUV PSF estimates is similar, and largely constrained to the core of these PSFs.

Table 2 and Figure 8 show that the PSF estimates have more energy in the wings than the diffraction-limited PSFs in both the NUV and the FUV channels. This indicates the instrument scatters more light than the diffraction-limited case. All else being equal, scattered light should be more prevalent at shorter wavelengths. However, our results show that scattering is less prevalent in the FUV than NUV, and scattering is a contributing factor to the reduction in PLSR in the NUV channel. We caution that the FUV transit data are of lesser quantity and quality than the NUV data. In particular, the FUV signal in the shadow of Mercury, which is critical to the estimation of scattered light, is comparatively weak and dominated by noise. Nevertheless, the result holds over the full range of our estimated uncertainties.

**Figure 9:**
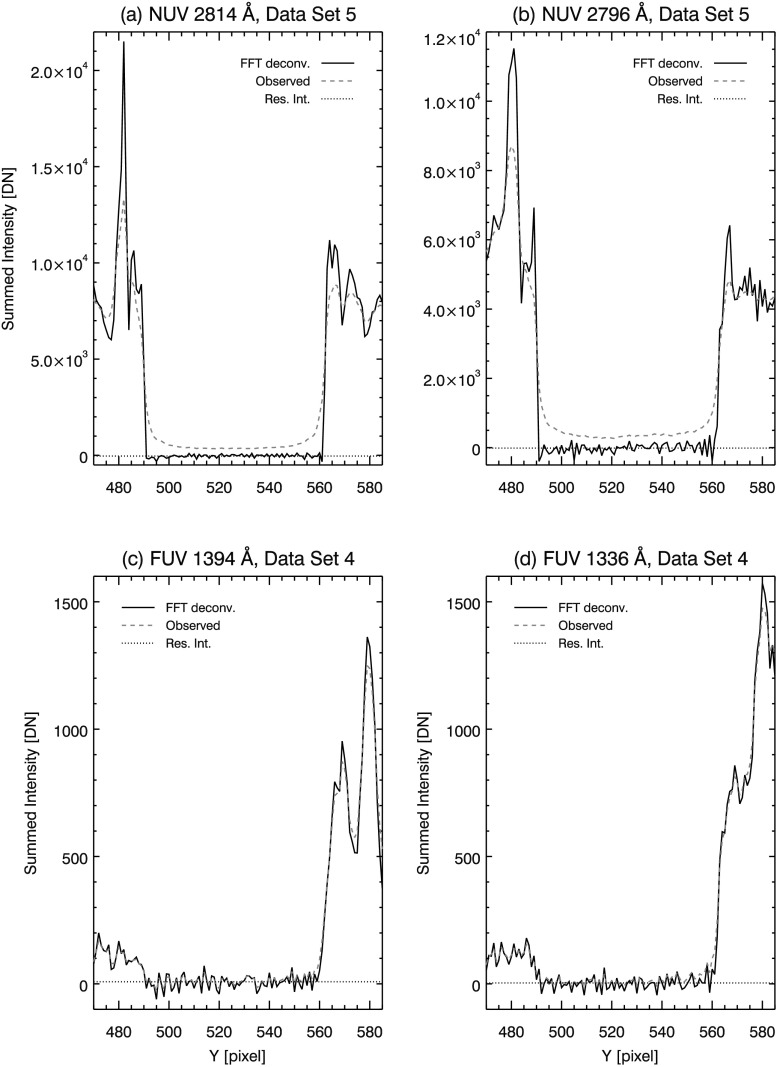
Deconvolution of reduced data using PSF estimates. Direct FFT deconvolution is used so that an unbiased estimate is obtained for the mean intensity in Mercury’s shadow (*dotted lines*). NUV 2814 Å (**a**) and 2796 Å (**b**) PSF estimates perform well, greatly sharpening the limb of Mercury (compare (a) and (b) in Figure 5) and reducing residual intensity to near zero. FUV 1394 Å (**c**) and 1336 Å (**d**) PSF estimates remove more residual intensity when compared to (c) and (d) of Figure 5.

To check the plausibility of the PSF estimates, we deconvolve them from the reduced data. By using Fourier deconvolution, we obtain an unbiased estimate of the residual intensity in Mercury’s shadow. Since we are using the best estimate (average) PSF on all the individual data sets, this is not merely a recapitulation of the cost function that was used as a stopping criterion in Section 3.3. Table 3 lists residual intensity in the shadow of Mercury after Fourier deconvolution. For the NUV and both FUV channels, we find that residual intensity after deconvolution is reduced to a fraction of a DN (mean, per pixel) in all but one case. The outlier in NUV data set 4 may result from small dark subtraction errors similar to those noted in Section 2.2. Figure 9 plots the results of Fourier deconvolution in solid curves for select reduced data sets. For comparison, the observed data is overplotted in dashed curves in each panel. The PSF estimates perform well in all data sets compared to the diffraction limit models in Figure 5; panels (a) – (d) in Figure 9 show that the limbs of Mercury are sharp and steep in the NUV and FUV channels, while residual intensity is reduced to near zero after deconvolution.

**Table 3:** Residual intensity in the shadow of Mercury after PSF deconvolution.

Data set	1336 Å		1394 Å		2796 Å^a^		2814 Å^a^	
	[DN]^b^	[%]^c^	[DN]^b^	[%]^c^	[DN]^b^	[%]^c^	[DN]^b^	[%]^c^
2					0.13	7.9	0.21	5.3
3					0.07	9.0	0.04	1.8
4	0.32	20.1	0.64	49.2	−1.84	−22.1	−1.49	−7.6
5					−0.06	−2.7	−0.37	−6.2
6	0.13	30.2	0.12	48.3	0.73	9.9	−0.65	−3.0
7					0.05	3.1	−0.13	−2.4
8	0.44	58.1	0.22	57.0	0.19	3.0	0.35	2.0
9a					−0.06	−3.6	−0.07	−1.6
9b					−0.05	−2.8	0.03	0.7
9c					−0.01	−0.6	0.04	0.9
9d					0.08	4.5	0.18	4.2
9e					0.05	2.8	0.19	4.3
9f					−0.11	−5.9	0.18	3.9

^a^Negative indicates residual intensity is less than zero after deconvolution.

^b^DN is the mean per-pixel value in the shadow of Mercury.

^c^Residual intensity given as percentage of original value before deconvolution.

**Figure 10:**
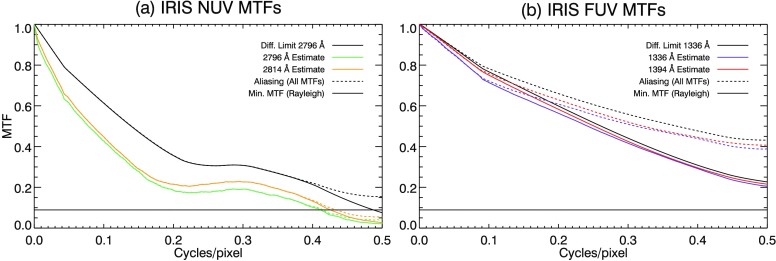
MTFs for the SG (**a**) NUV and (**b**) the FUV channels. Diffraction-limited performance (*solid black curve*) and resolution criterion (*dashed black curve*) are overplotted in both panels. A compensation has been applied to all MTFs to reduce the effects of aliasing (see text for details). Aliased curves are plotted in *dashed lines* for all MTFs.

The instrument resolution is estimated by the modulation transfer functions (MTFs) in Figure 10 panel (a) for NUV and (b) for FUV. In both panels of Figure 10 the diffraction-limited MTF is overplotted as a solid black curve. Aliasing (shown by the dashed curves in the figure) causes the MTF to be overestimated by double-counting at the Nyquist frequency. A compensation has been applied to reduce the effect of aliasing on each MTF (solid curves represent aliasing compensated results in Figure 10). The aliasing compensation is computed as follows. First a ‘non-aliased’ diffraction-limited PSF is modeled on a higher resolution grid, so that aliasing is negligible at the IRIS SG Nyquist frequency (refer to Section 3.1.2). We find that using 1024 pixels is sufficient to suppress aliasing, rather than the 420 pixel length of the reduced data sets. An aliased PSF is then created by applying a boxcar filter the width of an IRIS pixel to the non-aliased PSF. MTFs are then computed from both the aliased and the non-aliased PSFs. We define the *compensation curve* as the ratio of the non-aliased to the aliased diffraction-limited MTFs. The compensation curve is re-sampled to match the length of the reduced data set, then multiplied by the MTF estimates for each spectral window. The resulting compensated MTFs are reduced to half the value of the aliased versions at the Nyquist frequency.

We may estimate the spatial resolution of the IRIS SG by analogy to the Rayleigh criterion (Lord Rayleigh, 1879). We choose a resolution criterion of $9\%$, as this is the value of the MTF of the classic Airy pattern at the resolution attributed to it by Rayleigh. NUV MTFs are plotted in Figure 10(a), showing a significant degradation in MTF, dropping to almost a third of the diffraction limit at the Nyquist frequency. Despite the reduction in resolution, the 2796 Å MTF satisfies the resolution criteria out to 0.41 cycles/pixel (2.47 cycles/arcsec) and the 2814 Å MTF does slightly better at 0.42 cycles/pixel (2.55 cycles/arcsec). Panel (b) of Figure 10 shows that the MTFs for both FUV channels are reduced by ${\approx}\,10\%$ compared to the diffraction limit, but still double the resolution criterion at the Nyquist frequency. This shows that the FUV channels are essentially pixel-limited in resolution.

Our PSF estimates include subtle contributions from the blur induced geometric correction, as described in Section 3.1. As the geometric blurring varies in time and space, a PSF estimate and deconvolution that accounts in detail for the geometric correction would be prohibitively complicated. This complication could be sidestepped by deriving the PSF for Level 1 data, but that would be of limited value, since geometrical correction is desirable for nearly all scientific use. We have therefore worked from Level 2 IRIS spectra, which are the standard for scientific work. Since our method averages over the geometric blurring, we consider the resulting PSFs as the best average estimates that can be applied to any geometrically corrected IRIS spectra. However, we note that, for the same reason, we underestimate the *intrinsic* imaging capabilities of the instrument.

**Figure 11:**
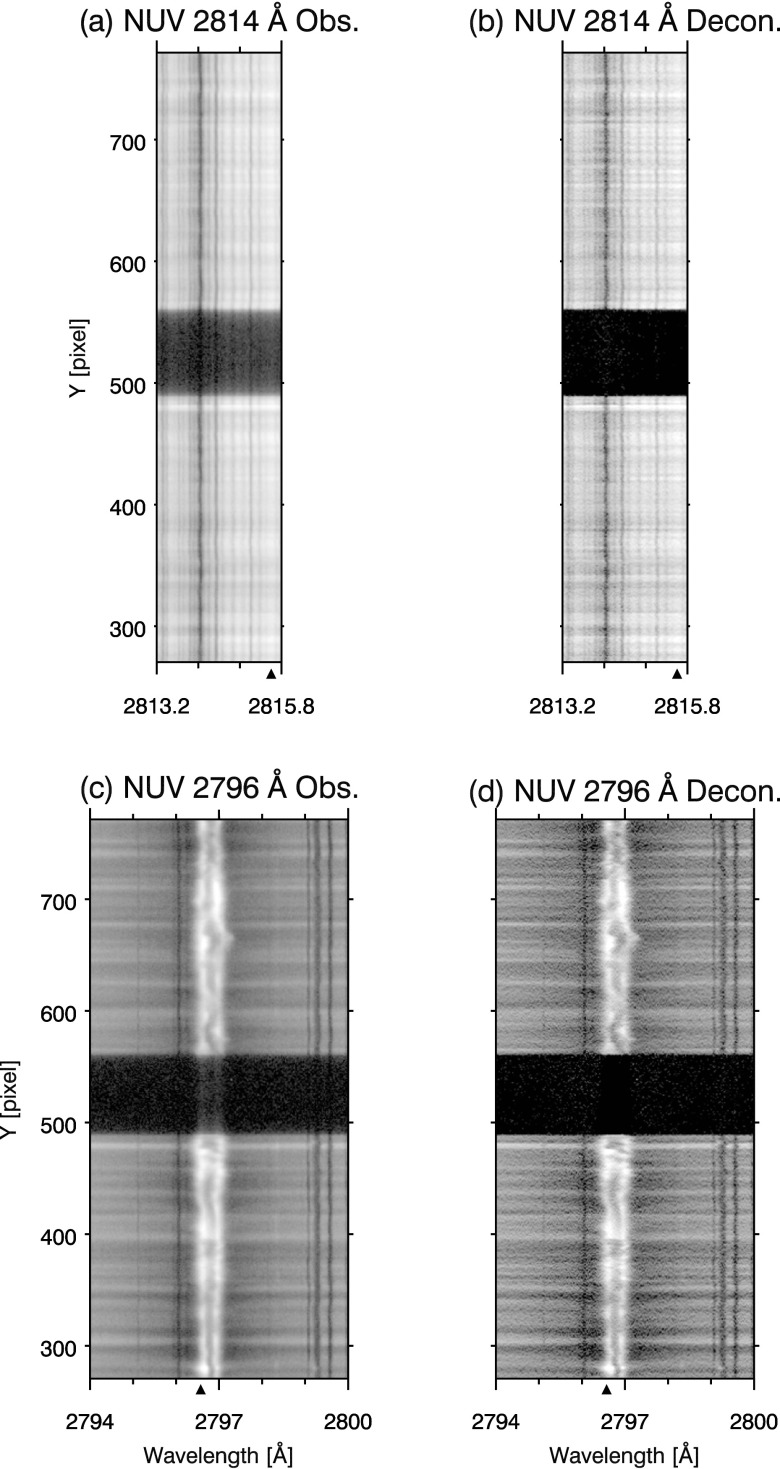
Comparison of original (**a**) and deconvolved (**b**) images for the IRIS FUV 2814 Å window. Remaining panels follow the same format: (**c**, **d**) 2796 Å. Spectra are square root scaled.

**Figure 12:**
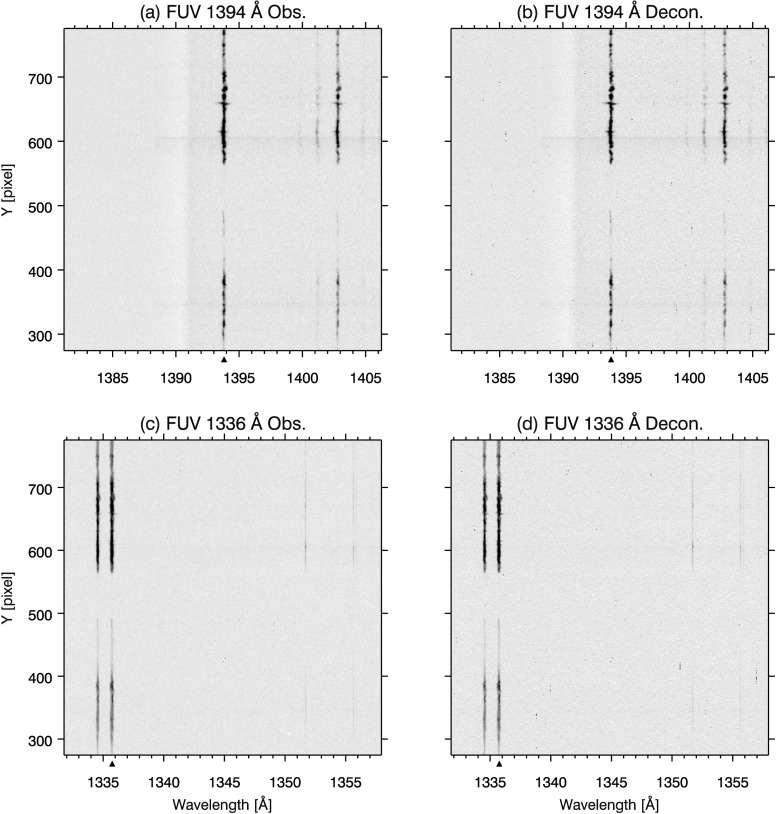
Comparison of original (**a**) and deconvolved (**b**) images for the IRIS FUV 1394 Å window. Remaining panels follow the same format: (**c**, **d**) 1336 Å. Intensity is displayed in inverse gray scale and logarithmically scaled.

**Figure 13:**
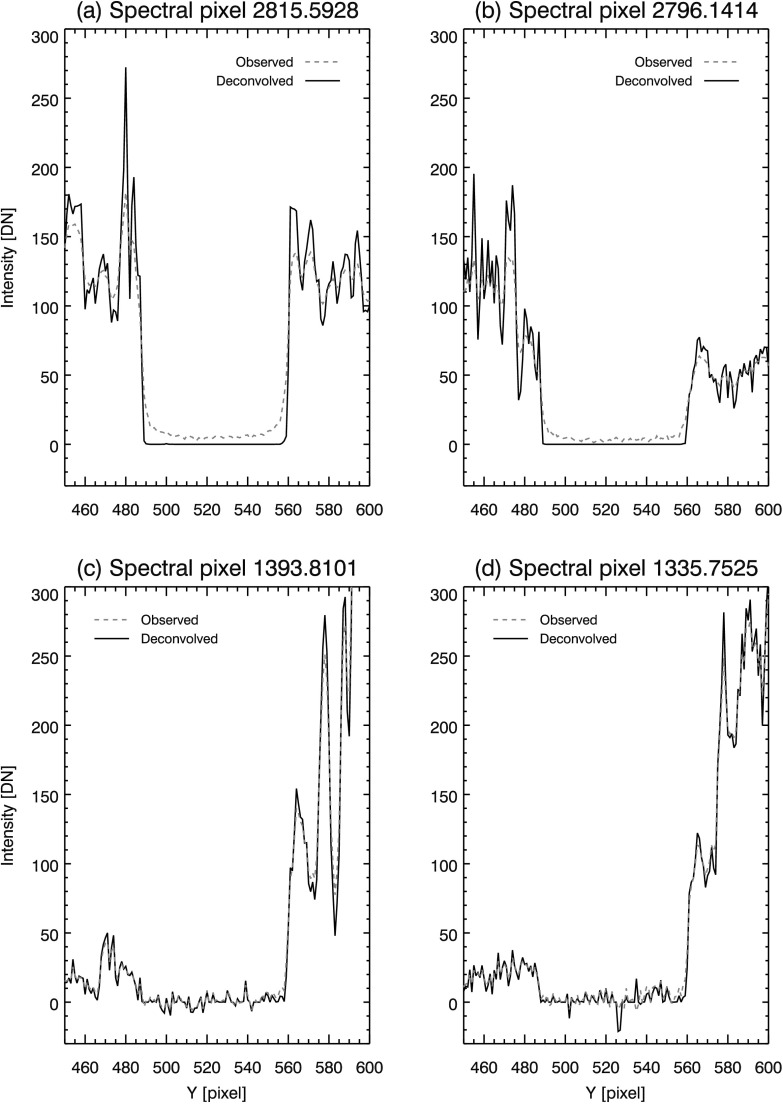
(**a**) Depicts intensity variations along a single spectral cut indicated by the black triangle on the wavelength axis in Figure 11(a) and (b). Panels (**b**), (**c**), and (**d**) show the same for Figure 11(c, d), and Figure 12(e, f), and (g, h), respectively.

In Figure 11, we present the results of deconvolution using our PSF estimates for the NUV 2814 Å (a, b) and 2796 Å (c, d). Figure 12 follows the same format for the FUV 1394 Å (a, b) and 1336 Å (c, d) spectral windows. The Mercury spectra from data sets 3 (FUV) and 4 (NUV) are used to illustrate the effect of PSF artifacts in Figures 11, 12, and 13. As the PSFs are one dimensional, deconvolution is performed only along the spatial axis. The deconvolved spectra are calculated by the Richardson–Lucy algorithm, using the same method and criteria described in Section 3.1.2. In both NUV spectra, contrast is enhanced throughout the images by deconvolution, evident in the narrower and more peaked spectrum of the deconvolved data in (a) and (b) of Figure 13. The limbs of Mercury are also sharpened to a steep cutoff (panels (a) and (b) in Figure 13). Scattered light, the ‘haze’ in the NUV spectra (a) and (c) of Figure 11, is eliminated in Mercury’s shadow in the corresponding deconvolved spectra (b) and (d).

The FUV channels show only modest improvement, since our FUV PSF estimates are essentially pixel limited. Scattered light is not apparent in either (a) or (c) of Figure 12 so that deconvolution in (b) and (d) has little to improve upon in this regard. Some sharpening of Mercury’s limbs is evident in panels (c) and (d) of Figure 13.

### Effect of the Initial Guess on the PSF

Our initial guess was a diffraction-limited PSF (Figure 4). To find how much this initial guess influences our results, we substituted a Voigt profile, fit to the diffraction-limited PSF, as the initializing PSF. A complete exploration of the initializing parameter space is beyond the scope of this document; we choose Voigt profiles as an alternate initialization since it is fundamentally different from the Fourier model PSFs. The initial Voigt profiles are smoother and lack the side lobes and undulating structure of the Fourier models.

**Figure 14:**
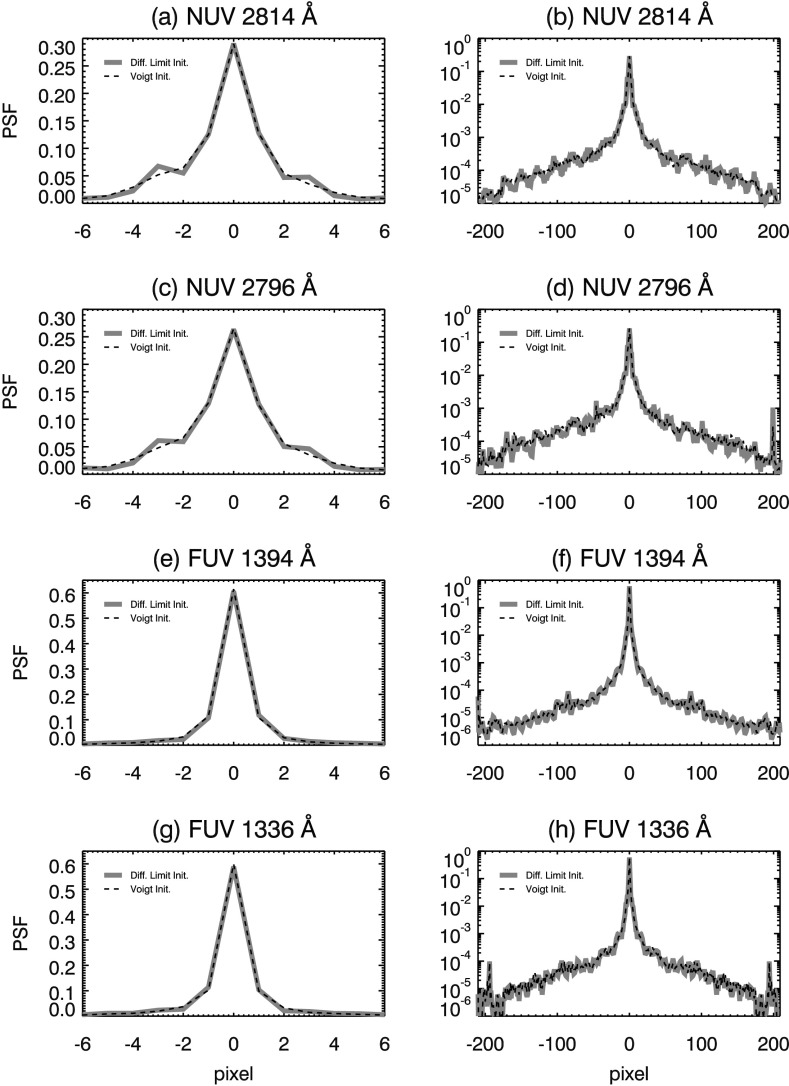
Comparison of results from an alternate Voigt PSF initialization (*gray curve*) to diffraction limited (*black curve*) for the four spectral windows. Both initializations result in prominent side lobes in the core of the (**a**) 2814 and (**c**) 2796 Å PSFs. For all PSFs derived from the Voigt initialization, the wings of the PSF are smoother; however, a distinct discontinuity appears at $\approx \pm 65$ pixels in panels (**b**), (**d**), and (**f**) and $\approx \pm 120$ pixels in panel (**h**).

PSF estimates from both initializations are compared in Figure 14; similar core structures and distinct, asymmetric side lobes result from either initialization (*i.e.* Figure 14(a) and (c)) for the NUV PSFs. We note that the side lobes and asymmetry appear in the NUV Voigt PSFs estimates, despite the absence of these traits in the initializing PSF. For all channels, the PSFs developed from the Voigt initialization have wings that are less noisy, and lack the undulations due to diffraction. This shows that our blind deconvolution method does not resolve fine structure far from the core of the PSF. Yet, the wings still fall off at similar rate compared to the diffraction-limited initialization. Subtle steps are visible in the wings in each NUV channel for the Voigt initialization (${\pm}\,65$ pixels panels (b) and (d) of Figure 14). Since these features appear in both NUV PSFs for this initialization, their presence may be genuine. They may also appear in the diffraction-initialized PSFs; however, their existence is somewhat obscured by undulations in the wing.

As a further diagnostic of initial conditions, we perform the deconvolutions in Section 4.2 using both the Fourier model and the Voigt initialized PSF. Side by side results from either initialization can then be compared. Hereafter we refer to the spectra obtained from the Voigt initialized PSF estimates as the alternate deconvolved spectra.

### PSF Effect on Data

**Figure 15:**
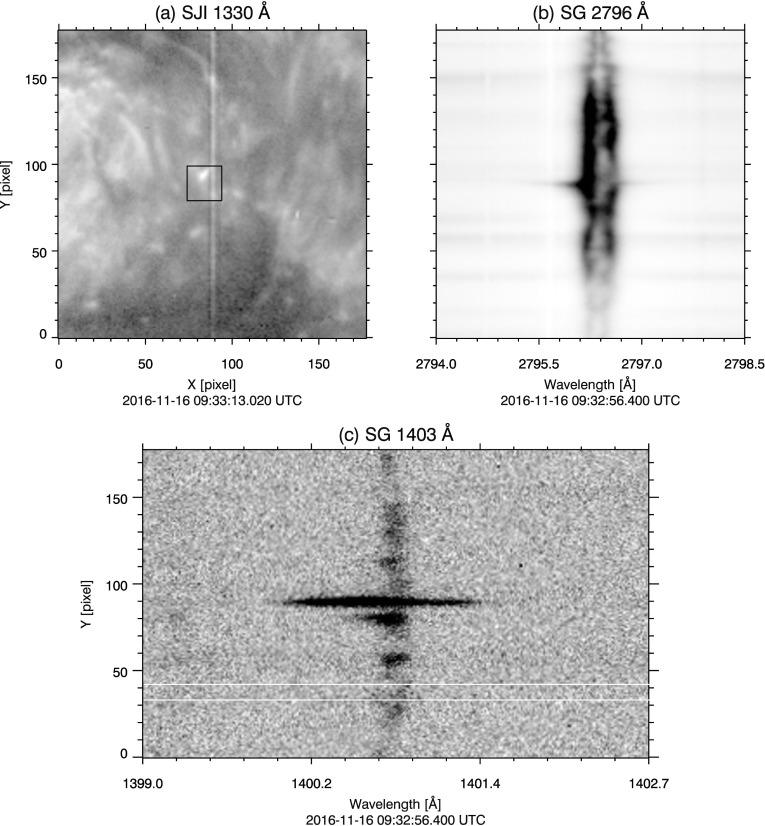
(**a**) IRIS SJI 1330 Å image of the explosive event used as a diagnostic of the SG PSFs. Boxed region outlines the approximate location of the EE (note that the SJI image was taken 17 s after the SG images). The EE results in a broadening of the spectra near Y pixel 89 in Mg ii (**b**) and Si iv (**c**) emission. *White horizontal lines* in (**c**) denote the region used for the FUV background subtraction.

To demonstrate the effect of the SG PSFs on IRIS observations, we consider a simple example of an explosive event (EE). Explosive events are ubiquitous, compact events characterized by large, non-thermal Doppler broadenings (${\approx }\,100~ \hbox{km}\,\hbox{s}^{-1}$ to the red or blue; Dere, Bartoe, and Brueckner, 1989; Dere, 1994) and may exhibit a bi-directional jet structure (Innes *et al.*, 1997). Typically EEs were observed in the FUV (*e.g.*, Si iv 1393 Å, and C iv 1548 and 1550 Å, Dere, Bartoe, and Brueckner, 1989; Innes *et al.*, 1997), but are also detected EUV and NUV wavelengths (*e.g.*, He ii 304 Å and Mg ii 2796 Å, Fox, Kankelborg, and Thomas, 2010; Huang *et al.*, 2014). Observations of bright, compact, and often isolated EEs are particularly sensitive to any PSF-induced blurring, and can serve as a diagnostic of the IRIS SG PSFs. For our example, we select a typical EE observed by IRIS close to disc center, shown in the boxed region of Figure 15(a). This event is well isolated from nearby bright features and is captured in only one frame in both the NUV and the FUV channels. Its Doppler signature is clearly visible in Mg ii (pixel 89 of panel (b), Figure 15) and particularly obvious in Si iv emission (panel (c), Figure 15). We use the 2796 Å and 1396 Å PSF estimates to deconvolve the spectra in Figure 15(b) and (c), respectively. Deconvolution is performed using the same method described in Section 3.1.2, again truncating after 50 iterations for the 2796 Å and 10 iterations for the 1403 Å window.

**Figure 16:**
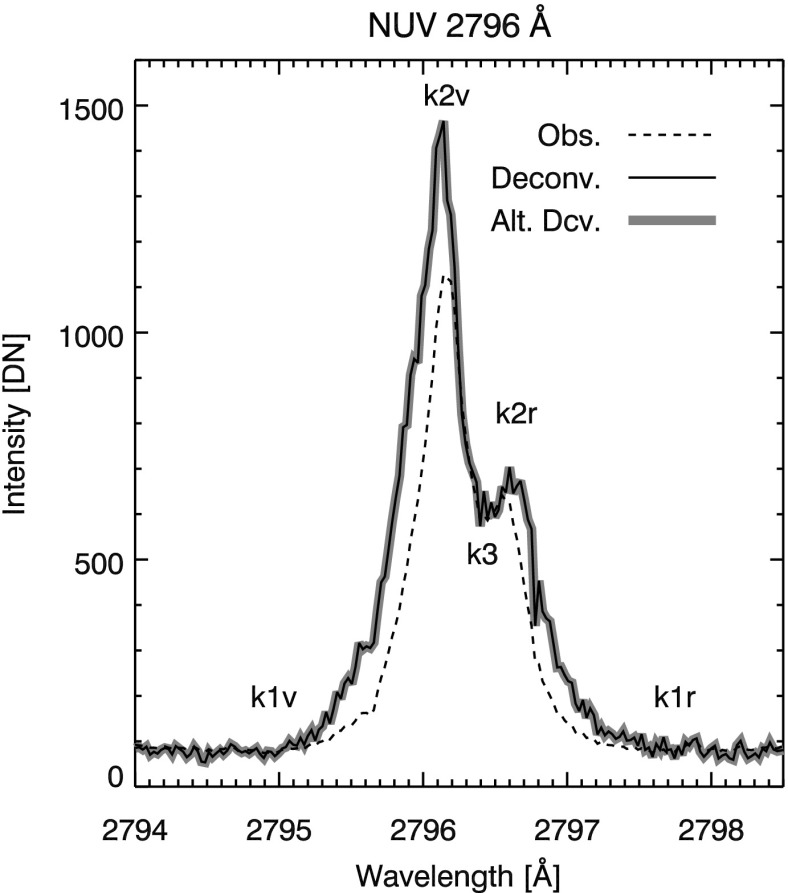
Mg ii k wing EE spectra.

The observed and deconvolved EE Mg ii k wing spectra is plotted in Figure 16. Labeling of the Mg ii profile follows convention, with features blueward of the line core ($k_{3}$) denoted by $v$ and redward by $r$. Leenaarts *et al.* (2013b) describe diagnostics of the upper chromosphere using the Mg ii h & k features, based on the formation properties discussed in detail in Leenaarts *et al.* (2013a). In Table 4 we re-list the Mg ii k spectral observables from Pereira *et al.* (2013), with the addition of the values observed from the NUV EE spectra for reference. The IDL routine iris_get_mg_features.pro (Pereira *et al.*, 2013) was used to derive the spectral observables for the EE in Table 4. If we were observing quiet Sun, the derived numerical values in Table 4 could be directly interpreted. However, we note that EEs are very dynamic and likely differ significantly from the plasma conditions in the quiescent Bifrost (Gudiksen *et al.*, 2011) simulations on which the results from Leenaarts *et al.* (2013a) and Leenaarts *et al.* (2013b) are based. We only use Table 4 to illustrate the quantitative impact of the deconvolution, and we do not mean to imply that the derived numerical values are necessarily correlated with the physical variables identified in Leenaarts *et al.* (2013b) and Pereira *et al.* (2013). Interpreted in this manner, Table 4 shows that deconvolution has significant impact on the diagnostic parameters of the Mg ii line profile.

**Table 4:** Correlation between Mg ii Features and Atmospheric Properties.

Spectral observable	Atmospheric property	Obs.	Deconv.	Alt. Dcv.
$\Delta v_{k3}$ ^a^	Upper chromospheric velocity	7.0	6.2	6.2
$\Delta v_{k2}$ ^a^	Mid chromospheric velocity	19.0	24.4	24.4
$\Delta v_{k3}-\Delta v_{h3}$ ^a^	Upper chromospheric velocity gradient	−1.1	−4.9	−4.9
*k* peak separation^a^	Mid chromospheric velocity gradient	39.4	50.0	50.0
$k_{2}$ peak intensity^b^	Chromospheric temperature	586.7	601.8	601.8
$(I_{k2v}-I_{k2r})/(I_{k2v}+I_{k2r})$ ^b^	Sign of velocity above *z*(*τ* = 1) of $k_{2}$	0.3	0.4	0.4

^a^[km s^−1^].

^b^[DN].

**Figure 17:**
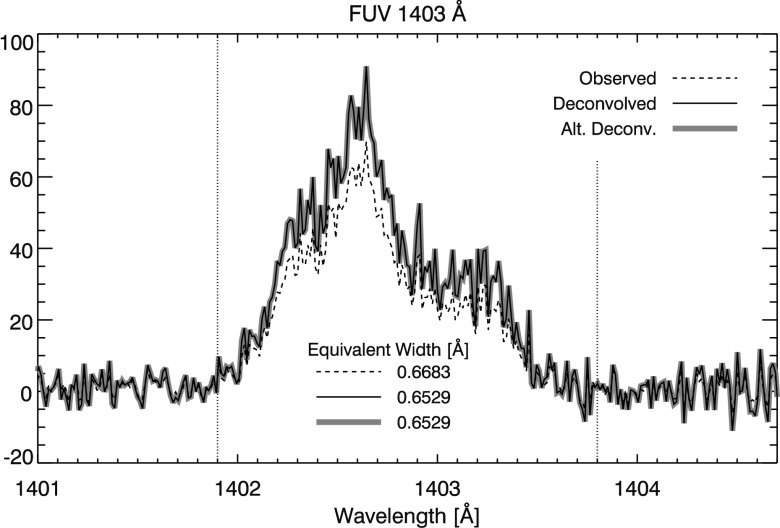
Si iv EE spectra. Equivalent width is calculated between *dotted vertical lines*.

Analysis of the Si iv EE spectra plotted in Figure 17 is more straightforward. The spectra are first background subtracted using the mean spectral value of the region bordered by the white horizontal lines in Figure 15(c) (the background is computed after deconvolution for the deconvolved spectra). The equivalent width is calculated between the vertical dotted lines in Figure 17, the approximate locations of where the shoulder of the spectral line goes to zero. This provides a direct measure of the spectral extent of the EE in each case. Since the FUV PSFs are very nearly diffraction limited, the effects of deconvolution are very subtle. In the example shown in Figure 17, equivalent width is only slightly reduced in the deconvolved spectra. We attribute the decrease in width to a combination of slightly increased fidelity in the deconvolved spectra and the introduction of noise from the deconvolution process.

Figures 16 and 17 and Table 4 all show that the Voigt and Fourier PSF initializations return identical results under deconvolution. We therefore conclude that the differences in the two PSF estimates in Figure 14 are below the level of accuracy that the underlying data can provide. We choose to provide the diffraction-limited PSF estimates in the associated SSW routine since these estimates are based on the physical properties of the instrument.

## Concluding Remarks

The PSF of the IRIS spectrograph results in non-zero intensity in the occulted area during a Mercury transit. From the Mercury transit spectra, we estimated instrument spatial PSFs for four spectral windows (2814 Å, 2796 Å, 1394 Å, and 1336 Å) of the NUV and FUV SG channels using a semi-blind Richardson–Lucy method.

In the NUV channel, we find similar PSFs in both the 2814 Å and the 2796 Å windows over multiple data sets. The NUV PSFs have broader cores and asymmetric side lobes, as well as broadened wings when compared to the diffraction-limited case (see, *e.g.*, Figures 4, 7, and 8). The pixel-limited Strehl ratio (PLSR) is 0.73 and 0.65 for the 2814 Å and 2796 Å windows, respectively. The PLSR is affected by increased energy in the side lobes and scattering wings of our PSF estimates. MTFs show that the NUV SG has a resolution (defined by $\mathrm{MTF}>0.9$) of 0.42 cycles/pixel (2.55 cycles/arcsec) and 0.41 cycles/pixel (2.47 cycles/arcsec) in the 2814 Å and 2796 Å windows, respectively.

For the FUV 1394 Å and 1336 Å windows, we estimate PSFs with 0.98 and 0.93 PLSR, respectively. The FUV MTFs are within 10% of their ideal values in both windows. The SG achieves Nyquist-limited resolution in both FUV channels.

We find that the IRIS SG PSFs contain more energy in the wings than the diffraction-limited PSFs in both the NUV and the FUV channels. We attribute this excess to scattering. Contrary to our expectations, scattering is apparently more prevalent in the NUV PSFs than in FUV.

Our results are derived from IRIS Level 2 SG data, which is blurred slightly by geometrical correction. We cannot reliably separate this contribution to our PSF estimates from intrinsic instrument effects. Therefore, we underestimate the true instrument performance.

Deconvolution of the PSF estimates improves the spectra in both FUV and NUV channels. Applied to the Mercury spectral data, we find that deconvolution reduces the residual intensity in Mercury’s shadow to a fraction of a DN in all channels, indicating that scattered light is reduced to near zero. NUV PSF artifacts, such as the blurring of Mercury’s limb, are also greatly reduced while contrast is enhanced, with no adverse effects noted. When applied to the spectra of an isolated EE, we find that deconvolution reveals significant differences in intensity and sometimes velocities derived from the Mg ii (NUV) line profiles. Deconvolution of the FUV EE spectra produces more subtle results, reducing the equivalent width of the line profile by 0.02 Å. In addition, the deconvolution can improve assessment of the physical size of detected features in both FUV and NUV channels.

The PSFs estimated here are distributed through SolarSoft (SSW) IDL, along with a program, iris_sg_deconvolve.pro, for direct application to IRIS level 2 data. PSFs for the 2814, 2796, 1394, and 1336 Å spectral windows are contained in the module, corresponding to the NUV and both FUV channels. The module performs a Richardson–Lucy deconvolution using the corresponding PSF estimate for each spectral window. The provided software will allow the community to perform more extensive testing of deconvolution of different types of solar scenes.
